# Non-invasive measurement of hemodynamic change during 8 MHz transcranial focused ultrasound stimulation using near-infrared spectroscopy

**DOI:** 10.1186/s12868-019-0493-9

**Published:** 2019-03-18

**Authors:** Evgenii Kim, Eloise Anguluan, Sangyeon Youn, Jihun Kim, Jae Youn Hwang, Jae Gwan Kim

**Affiliations:** 1 School of Electrical Engineering and Computer Science, Gwangju Institute of Science and Technology, Gwangju, 61005 Republic of Korea; 2 Department of Biomedical Science and Engineering, Gwangju Institute of Science and Technology, Gwangju, 61005 Republic of Korea; 3 Department of Information and Communication Engineering, Daegu Gyeongbuk Institute of Science and Technology, Daegu, 42988 Republic of Korea

**Keywords:** Near-infrared spectroscopy, Cerebral hemodynamics, Ultrasound brain stimulation

## Abstract

**Background:**

Transcranial focused ultrasound (tFUS) attracts wide attention in neuroscience as an effective noninvasive approach to modulate brain circuits. In spite of this, the effects of tFUS on the brain is still unclear, and further investigation is needed. The present study proposes to use near-infrared spectroscopy (NIRS) to observe cerebral hemodynamic change caused by tFUS in a noninvasive manner.

**Results:**

The results show a transient increase of oxyhemoglobin and decrease of deoxyhemoglobin concentration in the mouse model induced by ultrasound stimulation of the somatosensory cortex with a frequency of 8 MHz but not in sham. In addition, the amplitude of hemodynamics change can be related to the peak intensity of the acoustic wave.

**Conclusion:**

High frequency 8 MHz ultrasound was shown to induce hemodynamic changes measured using NIRS through the intact mouse head. The implementation of NIRS offers the possibility of investigating brain response noninvasively for different tFUS parameters through cerebral hemodynamic change.

## Background

Ultrasound is a widely used imaging tool for diagnosis and monitoring, which is gradually finding its way to therapeutic use. In particular, transcranial focused ultrasound (tFUS) has been demonstrated to be capable of modulating brain structures with considerable advantages over other neuromodulation techniques due to its combined noninvasive use, ability to penetrate deep brain structures, and precise targeting resolution [1, 2].

Ultrasound has already been implemented on animal models for neuroprotection after stroke [3], and in humans for neurological disorders [4], but the application of tFUS is still limited because of the incomplete understanding of its working mechanism and physiological effects on the brain [5]. Moreover, the method of selection of stimulation parameters that would specify the ability to either excite or suppress neural activity is also not determined, and thus further investigation is in demand [6].

Simulations modelling the human head have shown that the optimal frequencies for US transmission through the skull is below 0.7 MHz [7]. Higher frequencies may offer better spatial resolution but at the risk of increased attenuation and heating of the skull. For small animals however, the thinner skull reduces the effects of high frequency ultrasound absorption in the bone, potentially providing precise spatial stimulation specificity critical to animal studies. What remains is to determine whether the transmitted energy will be enough to induce brain activity in mice under safe sonication conditions.

Several studies measure physiological responses to assess the effects and to influence parameter selection for tFUS. The supporting techniques used in these studies have their strengths and weaknesses. The observation of a limb twitch along with electromyography is a common procedure to evaluate the effects of acoustic stimulation on brain circuits [8]. Even though the approach is straightforward, it fails to measure the immediate effects of tFUS in the brain region that do not have a quantifiable behavior change. On the other hand, more sophisticated techniques like functional magnetic resonance imaging (fMRI) [9] and positron emission tomography (PET) [10] are already well-established as valuable tools to observe indirect brain activity induced by tFUS, although they can be technically demanding, expensive, and with low temporal resolution; in addition to exposure to ionizing radiation in PET. In this work, we propose to use near-infrared spectroscopy (NIRS) as an alternative noninvasive approach that addresses the weakness of previous techniques to investigate the cerebral hemodynamic changes induced by tFUS.

In a manner similar to fMRI, NIRS can be applied to record changes in cerebral blood oxygenation related to brain activity during the execution of a specific paradigm. Although NIRS may not provide deep brain information, it is an attractive and accessible approach to investigate brain activity changes with its high temporal resolution, robustness to electrical artifacts, and low cost [11]. NIRS measures changes in intensity at different wavelengths, permitting estimation of cerebral hemoglobin concentration changes. The measure of change in oxygenated (HbO) and deoxygenated (RHb) hemoglobin could be used as an indirect way of recording brain activity due to the neurovascular coupling mechanism. NIRS has been used in various neuroscience studies including post-stroke rehabilitation [12], the anesthetic effect on the brain [13], functional brain connectivity [14], and investigation of other stimulation techniques [15]. We believe NIRS can be a suitable noninvasive tool to observe cerebral hemodynamic changes induced by tFUS as well as to identify the optimal stimulation parameters for a desired application. In this study, we aim to present the feasibility of NIRS to detect the brain hemodynamic change induced by different acoustic intensities with a central frequency of 8 MHz.

## Results

**Fig. 1:**
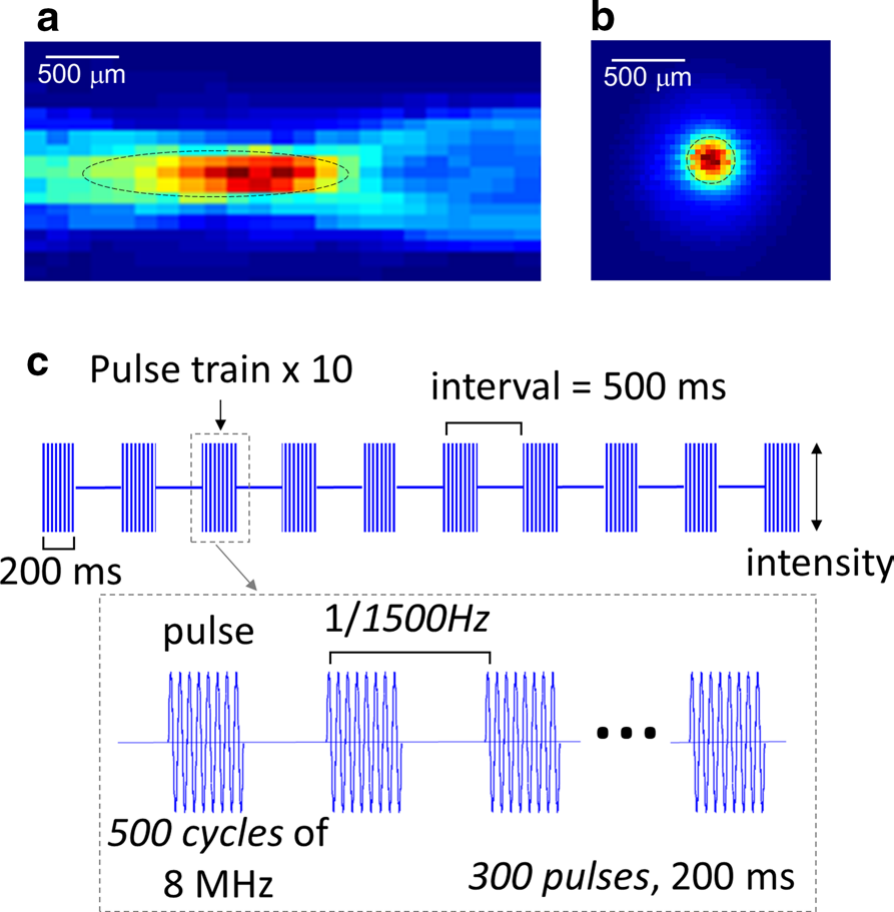
The ultrasound stimulation scheme. The beam profile for the 8 MHz transducer in the **a** lateral and **b** axial planes. **c** The 5 s stimulation consists of 10 pulse trains, each containing 300 pulses of 500 cycles of 8 MHz ultrasound with a PRF of 1500 Hz

The acoustic beam profile measured in water is shown on Fig. 1. The focal zone extends 1.76 mm in the lateral and 0.29 mm in the axial direction. Placing an excised mouse skull in between the transducer and hydrophone leads to an attenuation of 9 dB, maintaining 12% of the original acoustic power.

**Fig. 2:**
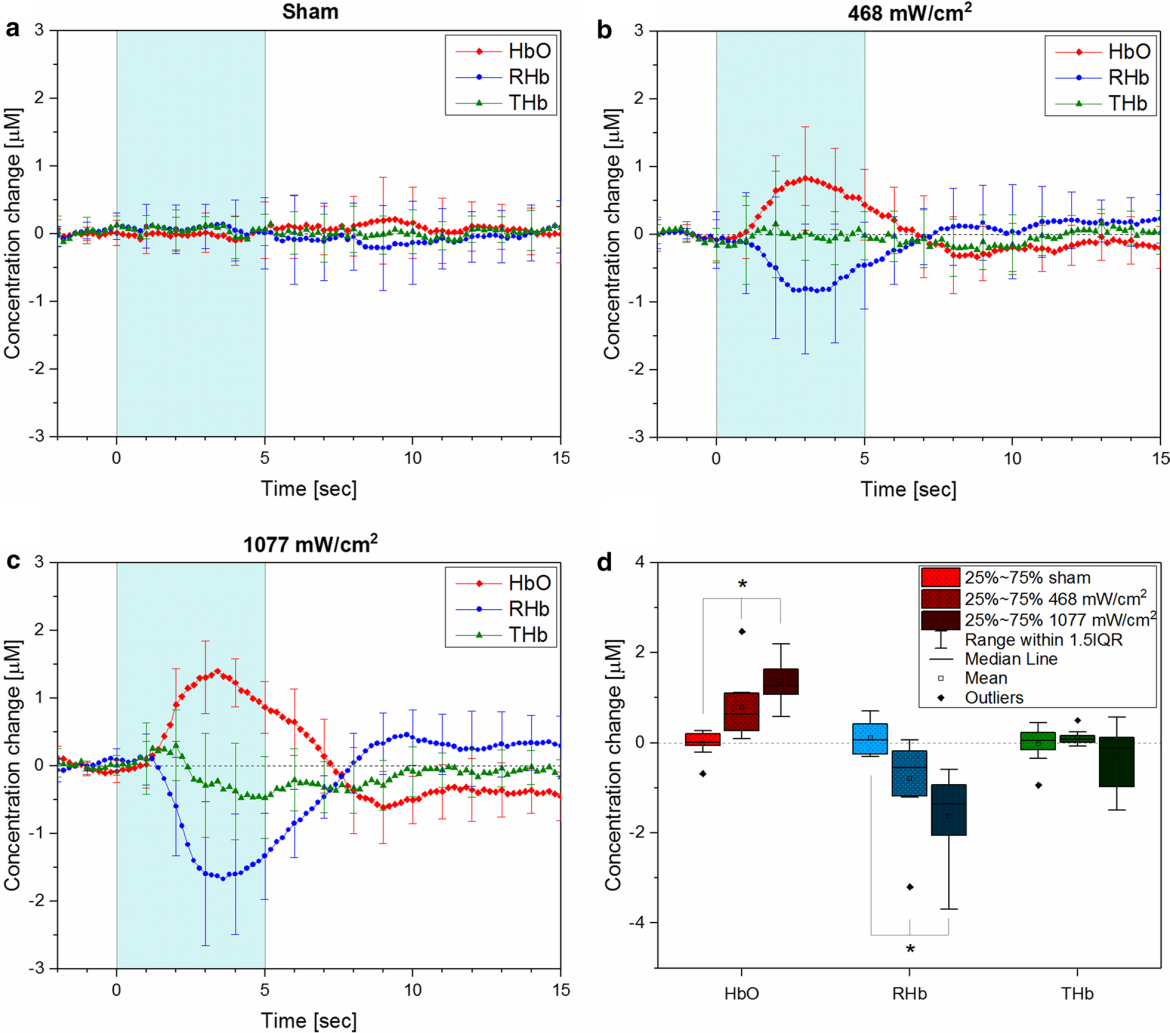
The averaged hemodynamic change from all the animals during various stimulation conditions. **a**–**c** The stimulation induced hemodynamic changes during and after the 5 s stimulation (shaded region). **d** The peak amplitude change in hemodynamic parameters for all stimulation conditions. One-way repeated measures ANOVA with Tukey–Kramer post hoc analysis (*p* < 0.05) of the averaged HbO, and RHb changes showed a statistically significant difference (*) in maximum amplitude depending on acoustic power

Cerebral hemodynamic changes were calculated from the NIRS signals obtained from 10 mice using the modified Beer–Lambert Law [16]. The 5 s ultrasound stimulation with repetition rate of 2 Hz (Fig. 1c) induced a change in cerebral hemodynamics as shown from the averaged results from all animals in Fig. 2a–c. The dramatic increase of HbO and decrease of RHb was consistently observed from all the mice during the stimulation but not in sham. Two simulation conditions with different acoustic intensities (I-_SPTA_ = 468 mW/cm^2^, 1077 mW/cm^2^) were delivered to the brain with the aim to observe the existence of any relationship between stimulation power and cerebral hemodynamic change. Increasing the acoustic intensity of stimulation also increases the observed hemodynamic changes in the mice. One-way repeated measures ANOVA show a statistically significant difference in maximum amplitude change of HbO (f(2, 18) = 19.9, *p* < 0.001) and RHb (f(2,18) = 15, *p* < 0.001), but not total hemoglobin THb (f(2,18) = 2.55, *p* = 0.1) between all three stimulation conditions. Tukey–Kramer post hoc analysis indicates that all three stimulation conditions produced hemodynamic profiles that are statistically distinct from each other (Fig. 2d). To assess the relationship between the hemodynamic profiles of the two stimulation conditions, a Pearson product-moment correlation was computed. Strong similarity for HbO and RHb was found with r^2^ > 0.9 for both.

## Discussion

The focus of this preliminary study was to determine if a reliable signal could be obtained with the simultaneous NIRS and tFUS. Although the fiber-based NIRS is difficult to implement with a larger number of channels due to the limited scalp space on a small animal model, it is still interesting due to the possibility of easy translation to human neuroimaging applications providing adequate spatial and temporal resolution [17].

A consistent pattern of cerebral hemodynamic change was observed across all animals tested during ultrasound stimulation. The results were in accordance with our previous report showing hemodynamic response initiated by tFUS using a minimally invasive neuroimaging technique [18]. In the previous study, wide-field cerebral hemodynamics was monitored using optical intrinsic signal imaging through an intact skull cranial window. Although the parameters and ultrasound frequency used are different from the current study, the stimulation evoked a similar pattern of hemodynamics as observed non-invasively through the scalp and skull using NIRS. Both acoustic stimulation protocols induced a pronounced increase and decrease of HbO and RHb respectively, a pattern that could be referred to as the general hemodynamic response to neural activity. The results also exhibited a post-stimulus undershoot similarly observed in the common BOLD response. The post-stimulus undershoot is presumed to be related to a prolonged consumption of oxygen [19] and known to be affected by stimulation conditions [20].

Although typically, the sub-megahertz frequency is recommended to use for tFUS due to the acoustic transparency of the human skull in this range, higher frequencies up to 43 MHz have been demonstrated to modulate neural excitation [21]. Higher frequencies offer better stimulation precision at the expense of attenuation through the skull. Therefore, finding a suitable frequency with sufficient transmission above 1 MHz may be more important for small animal studies. In a previous study, ultrasound with a frequency of 1.9 MHz has been shown to stimulate the motor cortex to produce repeatable contralateral limb movements in mice [22]. In addition, another research team demonstrated that the anatomical specificity of ultrasound stimulation to induce a tail response was greatly improved by using 5 MHz compared to 1 MHz [23]. However, since the motor response is limited to brain regions associated with inducing motion and can be affected by various parameters including the number of stimulated neurons, the absence of a motor response may not necessarily be related to the stimulation being insufficient to induce brain activity changes. In this work, high frequency 8 MHz tFUS applied to the somatosensory area with different acoustic intensities has been shown to be able to induce cerebral hemodynamic changes as probed by NIRS. Even with the observed changes in cerebral hemodynamics, no visible motor response was observed. Nonetheless, the mice still consistently showed motor response to fingers snapping due to the auditory startle reflex, in contrast with a recent study that elicited a startle response for both audible sound and low frequency ultrasound of 500 kHz [24]. The high acoustic frequency also provides an improvement in stimulation targeting in terms of focal size and minimizing the interference of reflected waves inside the skull [25, 26]. Focused sonication based on 8 MHz is highly absorbed by the mouse skull but, with an *f*–*number* of 1, can provide a focal diameter of approximately 200 um (1,540,000 m*s^−1^/8,000,000 Hz), that is about the same stimulation area for optogenetics based on a 200 um fiber [27]. Moreover, it affords the ability to obtain an adequate spatial resolution for imaging including functional ultrasound imaging, providing the possibility to implement a single ultrasound system for both neuromodulation and imaging.

In addition to central frequency, there are several important tFUS parameters that might affect the output of stimulation, among which is the peak acoustic intensity. The results using NIRS has shown the influence of acoustic intensity to the amplitude but not to the temporal profile of hemoglobin concentration change. The higher acoustic intensity induced a greater increase in cerebral blood oxygenation, which could be associated to an increase in neural activity and could play an important role in neuroprotection to minimize the effect of ischemic stroke [28].

Even though the obtained hemodynamic response agrees with the typical pattern induced by neural activity [29], the question of whether the obtained hemodynamic response originates from direct ultrasound neural stimulation or is only due to a cerebral vascular response is still unresolved. One possible solution would be the direct recording of neural activity through electrophysiology [30] or metabolic measurements using PET [31]. NIRS has also been shown to measure changes in the oxidation state of cytochrome-c-oxidase (oxCCO) which would indicate cellular energy metabolism [32]. However, the small concentration change of oxCCO compared to hemoglobin chromophores make the extraction of oxCCO a challenging task requiring additional computational complexity in the NIRS system [33].

The mechanical index of the applied ultrasound in this study (MI = 0.2) was much lower compared to the safety limit set for clinical ultrasound imaging applications (MI = 1.9), minimizing the likelihood of any cavitation-related brain tissue damage [34]. After passing through the skull, the acoustic intensities of the stimulation (I_SPTA_ = 129 and 56 mW/cm^2^) were also lower than the guidelines for ultrasound imaging (I_SPTA_ = 720 mW/cm^2^). Furthermore, previous reports with higher acoustic energy (I_SPTA_ = 6.4 W/cm^2^ in rabbit) did not show tissue damage [10]. The maximum temperature change of brain tissue was estimated to be less than 0.01 °C using previously described equations valid for short exposure times [35]. Obvious abnormalities in mouse behavior were not observed after stimulation.

## Conclusions

The work shows conceptually the ability of NIRS to noninvasively measure brain changes related to tFUS. The method is sensitive enough to show that the high frequency (8 MHz) causes the hemodynamic change, where the amplitude of cerebral hemodynamics are positively correlated to acoustic peak power. The authors believe that NIRS can be an important supplementary tool to investigate the effect of tFUS on cerebral hemodynamic change, as well as to find optimal acoustic parameters for a desired application.

## Methods

A total of ten female BALB/c mice weighing 17–20 g (9–10 weeks old) (Damul Science, Korea) were used for the experiment. The number of animals was determined using power analysis (power = 0.95, effect size = 1.3, η^2^ = 0.62 based on HbO data) [36]. The mice were housed in cages with 3–4 occupants at a 12-h light/dark cycle, having access to feed and water ad libitum. One day before the measurement, the mice were anesthetized using an intraperitoneal injection of ketamine-xylazine cocktail (80:10 mg/kg, respectively) to depilate the scalp. The animals were allowed to recover at least 24 h before the tFUS experiment.

Ultrasound stimulation was achieved using a focused single element ultrasound transducer with a focal distance of 19 mm, and 8 MHz fundamental frequency. The input sinusoidal pulses were generated from a function generator (Agilent 33220A, Keysight, USA) amplified at 50 dB (E&I 240L, USA). A 3D printed acoustic guide was attached to the end of the ultrasound transducer to provide convenient access to the mouse head. The ultrasound intensity and beam profile were measured from the tip of the waveguide in water and through the mouse skull using an acoustic intensity measurement system (AIMS III, ONDA, USA). The beam profile measured in water showed that the focus extends 1.76 mm in the axial and 0.29 mm in the lateral directions (Fig. 1a, b). Passing through the skull caused a 9 dB attenuation of the acoustic wave, keeping only 12% of the original power.

The continuous wave near-infrared spectroscopy setup consisted of two 400 µm optical fibers, 4 mm apart, positioned over the mouse scalp. One fiber was connected to a halogen broadband light source (HL-2000, Ocean Optics) and the other to a spectrometer (USB 4000, Ocean Optics), providing a single channel system. The 3D printed fiber holder provided additional restraint to the mouse head and optical fiber tips located at 0 mm anteroposterior (AP), and + 1 mm mediolateral (ML) for the light source fiber; and − 4 mm AP, + 1 mm ML for the detector fiber. The approximate position of the bregma was found by naked eye under sufficient illumination. Light intensity from 700 to 900 nm in 1 nm increments was converted to hemodynamic concentration change (HbO and RHb) using the modified Beer–Lambert law [16], Eq. 1, where SD is the separation distance between the source and detector, *ε* is the absorption coefficient, and Δ*A* is the difference in absorption between a time point and at a designated baseline (time t = 0). A mean optical pathlength at 720 nm was estimated by fitting the second derivative of the water feature at 720 nm and HbO feature at 760 nm [37] assuming a mean water content of 80% in the rodent brain [38]. The differential pathlength factor (DPF) at each wavelength was then derived by applying wavelength-dependent correction factors obtained from literature [39]. The THb concentration change is the sum of the obtained HbO and RHb. The processed data were low pass filtered at 0.1 Hz to reduce physiological high frequency respiratory and cardiac noises.

1 $$\left[ {\begin{array}{*{20}c} {\Delta [HbO]} \\ {\Delta [RHb]} \\ \end{array} } \right] = \frac{1}{SD}\left[ {\begin{array}{*{20}c} {\varepsilon_{HbO} (\lambda_{1} )DPF(\lambda_{1} )} & {\varepsilon_{RHb} (\lambda_{1} )DPF(\lambda_{1} )} \\ {\varepsilon_{HbO} (\lambda_{2} )DPF(\lambda_{2} )} & {\varepsilon_{RHb} (\lambda_{2} )DPF(\lambda_{2} )} \\ \vdots & \vdots \\ {\varepsilon_{HbO} (\lambda_{n} )DPF(\lambda_{n} )} & {\varepsilon_{RHb} (\lambda_{n} )DPF(\lambda_{n} )} \\ \end{array} } \right]^{ - 1} \left[ {\begin{array}{*{20}c} {\Delta A(\lambda_{1} )} \\ {\Delta A(\lambda_{2} )} \\ \vdots \\ {\Delta A(\lambda_{n} )} \\ \end{array} } \right]$$

Each animal was initially anesthetized with 3% isoflurane to restrain the animal in a stereotaxic frame. Once fixed, the animal was given at least 30 min to recover from anesthesia before the start of data acquisition. All animals received three different stimulation conditions, including sham, within a single acquisition experiment. The stimulation paradigms were selected in a way to keep the same pulse repetition frequency (PRF) of 1500 Hz and duty cycle of 9% but with different acoustic intensities (as measured in water, I_SPTA_) of 1077 and 468 mW/cm^2^. The acoustic parameters were chosen based on previous studies inducing a motor response for lower frequency ultrasound targeted to the motor cortex, and also considering sufficient acoustic power even after attenuation through the skull. The transmitted acoustic intensity (12%) is in a similar range with previously reported values for ultrasound brain stimulation in mice [40]. A diagram illustrating the pulse modulation for one pulse train is shown in Fig. 1c.

The experiment consisted of 75 randomly shuffled trials, with 25 trials for each acoustic condition. Each acquisition trial lasts for a total of 17 s including 5 s of stimulation containing ten ultrasound pulse trains with a repetition rate of 2 Hz. The interval between the start of each trial was fixed to 1 min. The ultrasound probe coupled with the acoustic guide was filled with acoustic gel and placed on the top of the mouse head pointing towards the somatosensory cortex. The ultrasound wave was focused along with the optical fibers for NIRS in such a way that the stimulated and monitored areas overlap. Sham trials were applied using the same protocol and setup as stimulation trials but without any input to the transducer. After the experiment, the mice were routinely observed for 24 h but no apparent behavioral change were detected. The mice were then euthanized using CO_2_ inhalation followed by cervical dislocation.

## Abbreviations

AP: anteroposterior
DPF: differential pathlength factor
fMRI: functional magnetic resonance imaging
HbO: oxygenated hemoglobin
ML: mediolateral
NIRS: near-infrared spectroscopy
oxCCO: oxidation state of cytochrome-c-oxidase
PET: positron emission tomography
PRF: pulse repetition frequency
RHb: deoxygenated hemoglobin
SD: separation distance
THb: total hemoglobin
tFUS: transcranial focused ultrasound
